# Supplementary material for: Don’t close the book on tocilizumab for the treatment of severe COVID-19 pneumonia–the jury is still out: The Kuwait experience
Source: PLoS One. 2021 Aug 24;16(8):e0254379. doi: 10.1371/journal.pone.0254379 (PMC8384154; doi:10.1371/journal.pone.0254379)
Supplement: Attachment — Submitted filename: Response to Reviewers.docx [file pone.0254379.s001.docx]

Response to reviewers

Thank you for giving us the opportunity to submit a revised draft of the manuscript “Don't Close the Book on Tocilizumab for the Treatment of Severe COVID-19 Pneumonia – the Jury Is Still Out: The Kuwait Experience” Cognitive Psychology of Metrical Constructs” for publication.

We appreciate the time and effort that you and the reviewers dedicated to providing feedback on our manuscript and are grateful for the insightful comments on and valuable improvements to our paper.

We have incorporated most of the suggestions made by the reviewers. Those changes are highlighted within the manuscript.

Reviewer #1: The data presented in this study is very helpful. It is a great addition to the portfolio of tocilizumab use for COVID-19.

Below are a few suggestions:
1. Title:
It would be better if the title follows a uniform pattern (title case). Below is a suggestion:
"Don't Close the Book on Tocilizumab for the Treatment of Severe COVID-19 Pneumonia – the Jury Is Still Out: The Kuwait Experience."

Author response: Thank you!

2. Abbreviations in the abstract and within the text:
- Add both the spelled-out version and the short form for SARS-CoV-2 and COVID-19 after one another in the abstract instead of having the abbreviations only
- Follow the same pattern in the introduction the first time these abbreviations are used (COVID-19, FDA, RT-PCR, etc.)
- Use the same naming pattern for all cytokines (full name and acronym when mentioning the name for the first time, and then use either the full name of the acronym, but not both or the acronym that was not listed next to the full name first)
- The same applies for using TCZ in the conclusion although the abbreviation was not mentioned before

Author response: Accordingly, throughout the manuscript, we have revised and edited all abbreviations and capitalizations.

3. Upper case:
There is no need to write tocilizumab and other medications, biomarkers, and other terms (such as liters) in upper case within the text.
Author response: Thank you! This has been addressed.
4. Discussion:
Comparing the mortality rate with previous studies is a great addition, but it would be better if the treatments of these studies are compared with the treatment given to this study subjects. If it is the same (hydroxychloroquine, Lopinavir-Ritonavir, and corticosteroid), then this should be mentioned too.

Author response: We agree with the reviewers, accordingly we have edited our conclusion and introduction to reflect such.

5. Conclusion:
- Write Parr et al. instead of Parr

6. English Language:
The article is well-structured, but it would be a good idea to seek independent editorial help to make the language clearer, error-free, and consistent.

Author response: Thank you, we have done our best to follow these comments.

Reviewer #2: In this study authors were reported the tocilizumab administration in patients with severe COVID-19. Guaraldi et al (https://doi.org/10.1016/S2665-9913(20)30173-9), Hernández-Mora et el (https://doi.org/10.1016/j.ijid.2020.10.045), Colaneri et al (10.3390/microorganisms8050695) and several previous studies reported similar findings.
he study is short for a original article (Short communication is suggested)
The title should be change to a more informative and the study. First paragraph of introduction is too general. 87.4% of the study population are male and it makes a concern in the results.

 Author response: Thank you, the introduction has been edited in an effort to be more concise and informative, new studies added includung RECOVERY, and REMAP-CAP trial.

Reviewer #3: PLOS ONE Review
Article title: Don't Close the Book on Tocilizumab for the Treatment of Severe
COVID-19 Pneumonia – The Jury is still out. The Kuwait Experience
Several concerns regarding the manuscript are described below:
Method
1. The authors used "Wilcoxon signed?rank test" to analyze the data. This test is designed for a paired group, such as pre-and post-treatment. But when we look up Table 2, this analysis seems not appropriate.

Author response: We have revised our statical analysis, and edited table 2 p values to reflect this. Thank you
Results
1. The author mentioned, "The mean age of the alive group was 54.3 years relative to the deceased group average age of 64.1 years." However, it should be noted that the deceased group was significantly older than the alive group. This should be explained as one of the possible factors for mortality and may be associated with chronic illnesses such as DM and hypertension.

Author response: Thank you for pointing this out. The reviewer is correct, and we have addressed this in the conclusion.
2. For age, it would be better to include standard deviation in Table 2.
3. For the outcome, is there data regarding the improvement of respiratory support after TCZ treatment?
4. Table 3. When there is no evidence of neutropenia, rash, anaphylaxis, why the percentage in table 3 were 100%?

Author response: Thank you for pointing this out. We have edited Table 3 to reflect the correct results, and omitted neutropenia, rash, anaphylaxis.

Discussion
1. The authors mentioned, "Consistent with previous studies, the deceased group were predominantly older males who tended to have…..". However, there is no explanation or data to support this statement in the results section, and all of a sudden, it was described in the discussion.
2. Since TCZ is an immunosuppressive agent, it is not surprising that secondary infection is observed in both groups. Still, I think the authors need to add an additional explanation for this matter.

Author response: Thank you for your feedback, we have attempted to reconstruct the conclusion to reflect your comments.
